# A Rare Case of Splenic Marginal Zone B-Cell Lymphoma Mimicking Relapsing Polychondritis of the Ear

**DOI:** 10.1155/2014/139386

**Published:** 2014-12-02

**Authors:** Gary J. Huang, Bryan Mendes, Kianoush Sheykholeslami

**Affiliations:** ^1^OSF Saint Anthony Health System, Department of Surgery, University of Illinois College of Medicine at Rockford, Rockford, IL 61103, USA; ^2^OSF Specialty Clinic, 698 Featherstone Road, Guilford Square, Rockford, IL 61107, USA; ^3^OSF Health System, Section of Otolaryngology, Head and Neck Surgery, Department of General Surgery, University of Illinois College of Medicine at Rockford, Rockford, IL 61107, USA; ^4^Department of Biomedical Science, University of Illinois College of Medicine at Rockford, Rockford, IL 61101, USA

## Abstract

Relapsing polychondritis (RPC) is a poorly understood phenomenon associated with cartilaginous inflammation of the ear, nose, tracheobronchial tree, and peripheral joints. Many cases of RPC respond to anti-inflammatories and resolve with no further complications. However, RPC has also been linked to more insidious conditions such as malignancies, autoimmune disorders, vasculitis, or underlying infections. Given the spectrum of associated disorders, patients with RPC may need to be monitored for more insidious underlying conditions. In this case, we report a unique case of bilateral auricular inflammation and nasal inflammation mimicking RPC as the only presenting symptom of splenic marginal zone B-cell lymphoma and we survey related cases in the literature.

## 1. Introduction

Auricular inflammation has a broad differential, including atopic dermatitis, contact dermatitis, cellulitis, psoriasis, systemic lupus erythematosus (SLE), other autoimmune cartilaginous conditions, chondrodermatitis nodularis, malignancy, trauma, or relapsing polychondritis (RPC). Diagnosis is typically rendered by clinical signs and symptoms but can require biopsy for a definitive tissue diagnosis.

Relapsing polychondritis is a disorder of unknown etiology first described in 1960 that causes systemic inflammation and destruction of cartilage. The incidence is unclear, but Luthra [1] reported an incidence of 3.5 cases per million in one city in 2000. RPC can affect the nose, peripheral joints, tracheobronchial tree, and eye and cause other nonspecific skin lesions.

RPC has been theorized to be an autoimmune condition targeted against cartilage proteoglycans or possibly a paraneoplastic effect. In fact, up to fourth of patients with RPC may have an underlying myelodysplastic syndrome [2]. RPC is diagnosed clinically, but elevated inflammatory markers, anemia, and leukocytosis can be helpful in gauging disease activity. The McAdam et al. [3] criteria that were originally used to diagnose RPC have been modified several times since inception, but diagnosis generally relies on a combination of bilateral auricular chondritis, nonerosive inflammatory polyarthritis, nasal chondritis, ocular inflammation, respiratory tract chondritis, and vestibular dysfunction. Histological diagnosis can also be helpful and is used in the Damiani and Levine criteria [4].

Malignancy involving the ear auricles also presents as inflammation but rarely is it bilateral. We present an unusual case of RPC-like symptoms that were diagnosed as splenic marginal zone lymphoma (SMZL) with cutaneous manifestations. SMZL is a neoplasm of small B-lymphocytes that replaces white pulp germinal centers in the spleen. SMZL is relatively rare, constituting less than 1% of all non-Hodgkins lymphomas [5]. It occurs almost exclusively in patients over 50 years of age, with median age of presentation at 65 years. Patients with SMZL typically present with splenomegaly, lymphocytosis, and cytopenia due to hypersplenism [6, 7]. SMZL does not typically have lymphadenopathy, systemic symptoms, constitutional “B” symptoms, or extra-lymphatic involvement. The incidence is twice as high in patients of Caucasian ancestry as other races, with no gender predominance.

The prognosis is generally excellent, with median overall survival in excess of 10 years. However, there is a subset of SMZL that is extremely aggressive with a median survival of 18 months [8]. Treatment is controversial, as marginal zone lymphomas are relatively rare and there are few randomized trials comparing treatments.

Given the retrospective nature of this study, it was granted an exemption in writing by the Institutional Review Board (IRB) committee of OSF Saint Anthony Medical Center.

## 2. Case Report

A 71-year-old Caucasian male initially presented to his primary care provider with nontender erythema of his right ear. The patient denied any trauma, fever, chills, diaphoresis, hearing involvement, weight loss, or otorrhea. Past medical history was significant for aortic and mitral valve replacements, atrial fibrillation treated with Coumadin, and chronic splenomegaly. The patient was placed on a 20 mg prednisone taper, which failed to resolve the erythema or rash after 2 weeks. In fact, there was found to be bilateral ear involvement, nasal involvement, and new onset tenderness at the 2-week follow-up. Patient was placed on 0.1% Triamcinolone EX CREA for suspected polychondritis with orders for erythrocyte sedimentation rate (ESR), anti-nuclear antibody (ANA), and rheumatoid factor tests. ESR was significant at 101 mm/h (normal 0–15 mm/h), ANA was negative at a 1 : 80 dilution, and RF QT was negative at <15 IU/mL.

**Figure 1:**
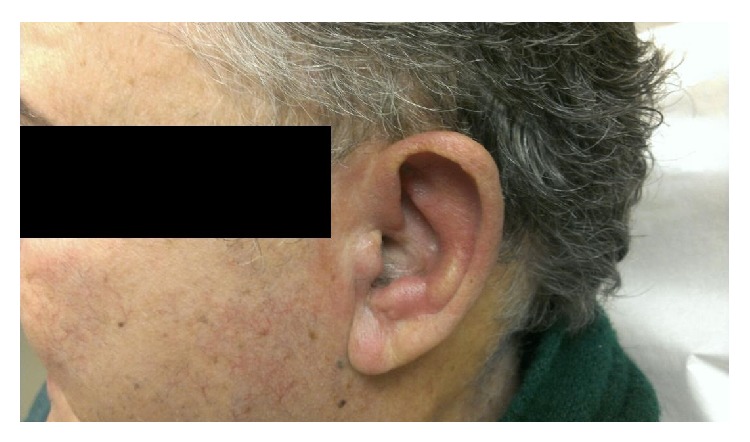
Left lateral view, initial visit.

**Figure 2:**
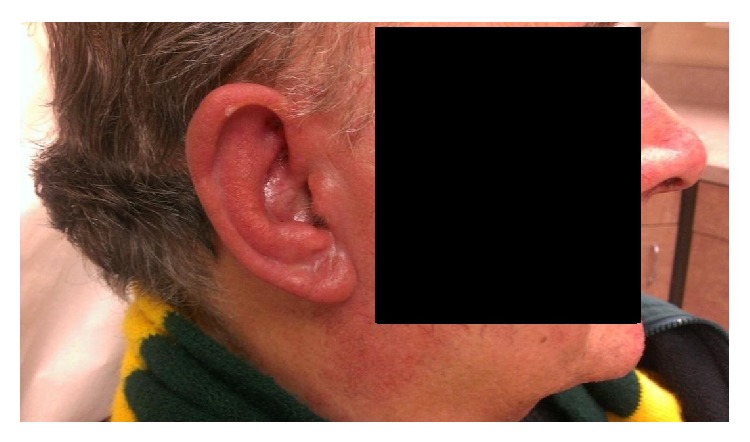
Right lateral view, initial visit.

Patient was referred to otolaryngology for evaluation. Physical examination was significant for erythematous, edematous, tender auricles bilaterally (Figures 1 and 2), and nasal tip with unremarkable findings on the rest of the exam. The left auricle had the classic lobule-sparing inflammation of RPC, whereas the right auricle presented with tenderness of the entire ear. A punch biopsy was performed due to clinical suspicion for relapsing polychondritis.

**Figure 3:**
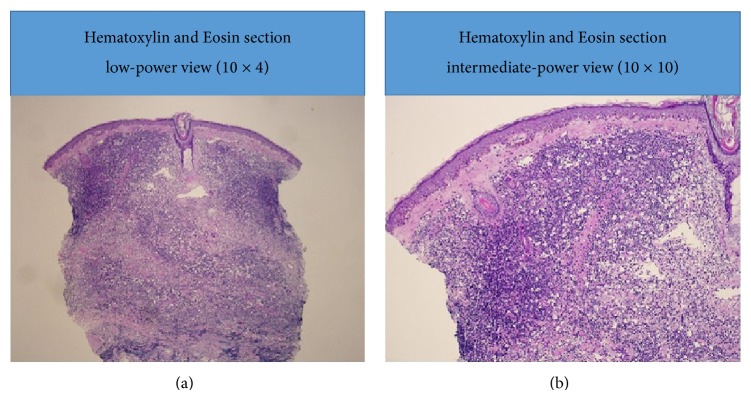
Hematoxylin and Eosin stain, light microscopy 10 × 4 (a), 10 × 10 (b).

**Figure 4:**
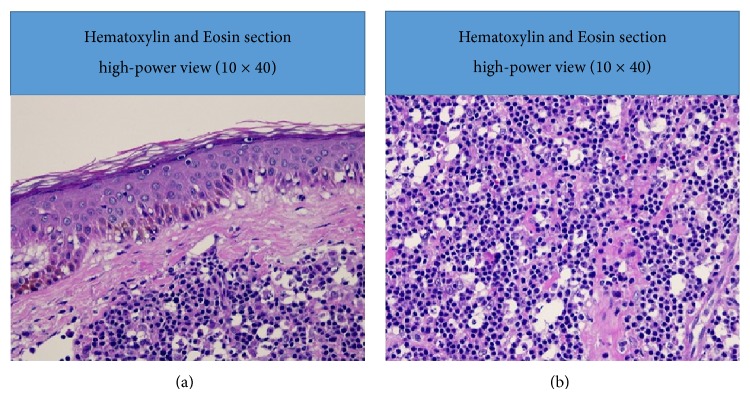
Hematoxylin and Eosin stain, light microscopy 10 × 40 (a), 10 × 40 (b).

**Figure 5:**
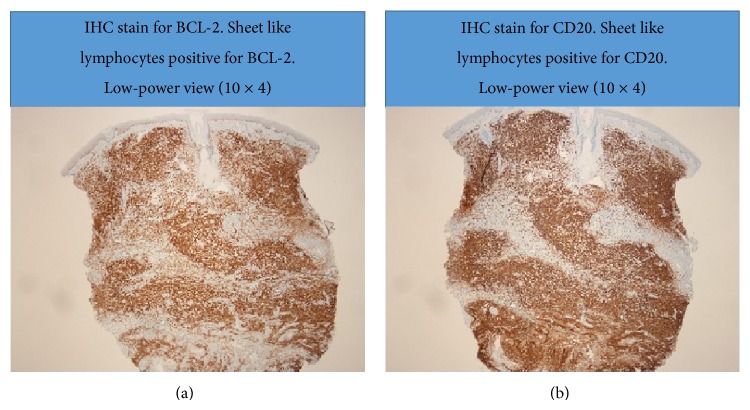
(a) Immunohistochemistry stain for BCL-2, light microscopy 10 × 4. (b) Immunohistochemistry stain for CD20, light microscopy 10 × 4.

**Figure 6:**
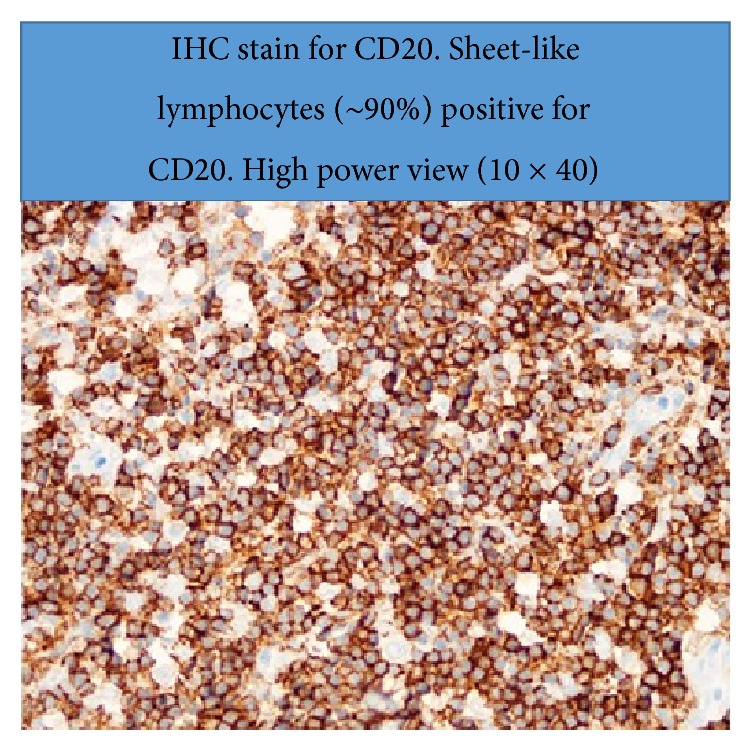
Immunohistochemistry stain for CD20, light microscopy 10 × 4.

Surgical pathology (Figures 3, 4, 5, and 6) showed atypical diffuse lymphoid infiltrate of the superficial and deep dermis favoring low grade B-cell lymphoma, marginal zone subtype. Ki-67 proliferation index showed 10% of tumor cells with positive nuclear stain. Immunohistochemistry staining was equivocal for CD20, BCL-2, and CD45. Staining was negative for CD3, CD5, CD10, CD23, CD30, and cyclin D1. Outside consultation agreed with the diagnosis. Clinical correlation by hematology and oncology indicated a diagnosis of likely primary cutaneous marginal zone lymphoma (PCMZL) with plasmacytic differentiation. Oncology ordered a colonoscopy, esophagogastroduodenoscopy, staging CT scan of the chest and pelvis, and a trial of Rituxin (Rituximab).

Complete blood count (CBC) showed a normocytic anemia (hemoglobin level of 9.8 g/dL) with white blood cell count and platelets within normal limits. CT of the chest, abdomen, and pelvis showed slightly increased splenomegaly from prior CT and minimally enlarged retroperitoneal lymph nodes. There were no other changes from a baseline CT performed 4 months prior to admission. Esophagogastroduodenoscopy was unremarkable, and biopsies showed no abnormalities. Colonoscopy showed a single nonbleeding vascular ectasia and diverticulosis with no other abnormalities. Patient was then referred to a tertiary care center for further evaluation. Of note, the patient's auricular inflammation and tenderness resolved between oncology evaluation and referral to the tertiary care center. Lactate dehydrogenase (LDH), C-reactive peptide (CRP), bone marrow biopsies, and screens for hepatitis B, hepatitis C, and HIV were performed. LDH was elevated at 279 (range 122–222 U/L) and CRP was elevated at 63.6 mg/L (normal <8.0 mg/L). Hepatitis B, hepatitis C, and HIV screens were negative. Bone marrow biopsy showed hypercellular marrow with involvement by the lymphoma, constituting approximately 10–15% of the total marrow cellularity.

A splenectomy and liver biopsy were subsequently performed. Splenectomy specimen showed splenic marginal zone lymphoma with involvement of the spleen and hilar lymph nodes. Specimen was CD5+, CD20+, and weakly CD19+. Immunohistochemistry showed no reactivity for CD10, CD23, CD3, CD7, and CD10. Patient was assessed to not require further treatments after splenectomy and would be closely followed at the tertiary center. To our knowledge, the patient has not had a recurrence of his RPC-like symptoms.

## 3. Discussion

Given the undefined etiology of relapsing polychondritis and the potential connection to malignancy, providers must be vigilant in evaluating patients, particularly in cases where RPC is refractory to initial treatment. Miller et al. [9] described the first case of malignancy presenting as relapsing polychondritis in 1974, and there have been several reports of malignancies presenting as RPC since then. RPC has a strong association with leukemia and lymphomas in particular. Fransen et al. [10] reported a case of chondrosarcoma that initially presented as costochondritis, auricular inflammation, and inflammatory polyarthritis. Bochtler et al. [11] reported a case of chronic lymphocytic leukemia that presented as inflammatory polyarthritis, ocular inflammation, and bilateral auricular chondritis. Castrejon et al. [12] reported a case of unilateral left auricular inflammation, uveitis, and erythema nodosum diagnosed as lymphoplasmocytic lymphoma. Lichauco et al. [13] reported a case of MALT lymphoma presenting as bilateral auricular thickening, but also with proptosis and polyarthralgia. It is evident from the literature that the constellation of symptoms in RPC may indicate a more insidious underlying systemic disease.

The treatment of SMZL is currently unclear. Initially, splenectomy was considered the first line treatment as it improved cytopenia, lymphocytosis, and progression free survival (PFS) [14]. Updated follow-up in 2013 also confirmed the benefits of splenectomy [15]. In addition, splenectomy has palliative effects on abdominal discomfort and prolonged the length of time before remission [16]. However, the overall survival, risk of histological transformation, and risk of death did not change with splenectomy. Other studies, in fact suggest that there is no difference in outcomes with or without splenectomy [16]. Rituximab, an anti-CD20 monoclonal antibody, has efficacy and complication rates equivalent to splenectomy and is an option in patients where surgery is relatively contraindicated. Milosevic et al. [17] found that patients treated with combination splenectomy and chemotherapy improved survival and disease remission when compared to splenectomy alone. Two studies found that chemotherapy with Rituximab was an effective treatment option for SMZL, superior to splenectomy, and supported the idea that splenectomy along with chemotherapy was more efficacious in inducing remission and improving survival than splenectomy alone [18, 19]. However, given the rarity of SMZL and the paucity of robust data, providers must tailor treatments to individual patients.

This is the first ever reported case of SMZL with a presentation mimicking relapsing polychondritis. The case reinforces the idea that physicians treating relapsing polychondritis must be vigilant in searching for underlying malignancies and myelodysplastic syndromes such as B-cell lymphoma, particularly when the condition does not respond to standard treatment.

## References

[1] Luthra H. S., Klippel J. H., Dieppe P. A. (2000). Relapsing polychondritis. *Rheumatology*.

[2] Yanagi T., Matsumura T., Kamekura R., Sasaki N., Hashino S. (2007). Relapsing polychondritis and malignant lymphoma: is polychondritis paraneoplastic?. *Archives of Dermatology*.

[3] McAdam L. P., O'Hanlan M. A., Bluestone R., Pearson C. M. (1976). Relapsing polychondritis: prospective study of 23 patients and a review of the literature. *Medicine (Baltimore)*.

[4] Damiani J. M., Levine H. L. (1979). Relapsing polychondritis—report of ten cases. *Laryngoscope*.

[5] Liu L., Wang H., Chen Y., Rustveld L., Liu G., Du X. L. (2013). Splenic marginal zone lymphoma: a population-based study on the 2001–2008 incidence and survival in the United States. *Leukemia and Lymphoma*.

[6] Audouin J., Le Tourneau A., Molina T., Camilleri-Broët S., Adida C., Comperat E., Benattar L., Delmer A., Devidas A., Rio B., Diebold J. (2003). Patterns of bone marrow involvement in 58 patients presenting primary splenic marginal zone lymphoma with or without circulating villous lymphocytes. *British Journal of Haematology*.

[7] Franco V., Florena A. M., Iannitto E. (2003). Splenic marginal zone lymphoma. *Blood*.

[8] Chacón J. I., Mollejo M., Muñoz E., Algara P., Mateo M., Lopez L., Andrade J., Carbonero I. G., Martínez B., Piris M. A., Cruz M. A. (2002). Splenic marginal zone lymphoma: clinical characteristics and prognostic factors in a series of 60 patients. *Blood*.

[9] Miller S. B., Donlan C. J., Roth S. B. (1974). Hodgkin's disease presenting as relapsing polychondritis. A previously undescribed association. *Arthritis and Rheumatism*.

[10] Fransen H. R. A., Ramon F. A., De Schepper A. M. A., De Beuckeleer L. H. L., Bal J., Goovaerts G. (1995). Chondrosarcoma in a patient with relapsing polychondritis. *Skeletal Radiology*.

[11] Bochtler T., Hensel M., Lorenz H.-M., Ho A. D., Mahlknecht U. (2005). Chronic lymphocytic leukaemia and concomitant relapsing polychondritis: a report on one treatment for the combined manifestation of two diseases. *Rheumatology*.

[12] Castrejon I., Ibanez M., Vicente E., Steegmann J. L., Castaneda Sanz S. (2007). Relapsing polychondritis associated with a lymphoplasmocytic lymphoma and erythema nodosum. *Reumatologia Clinica*.

[13] Lichauco J. J., Lauer S., Shigemitsu H. H. (2001). Orbital mucosa-associated lymphoid tissue (MALT)-type lymphoma in a patient with relapsing polychondritis. *Arthritis & Rheumatology*.

[14] Thieblemont C., Felman P., Berger F., Dumontet C., Arnaud P., Hequet O., Arcache J., Callet-Bauchu E., Salles G., Coeffier B. (2002). Treatment of splenic marginal zone B-cell lymphoma: an analysis of 81 patients. *Clinical Lymphoma*.

[15] Lenglet J., Traullé C., Mounier N. (2014). Long-term follow-up analysis of 100 patients with splenic marginal zone lymphoma treated with splenectomy as first-line treatment. *Leukemia & Lymphoma*.

[16] Olszewski A. J. (2012). Survival outcomes with and without splenectomy in splenic marginal zone lymphoma. *The American Journal of Hematology*.

[17] Milosevic R., Todorovic M., Balint B., Jevtic M., Krstic M., Ristanovic E., Antonijevic N., Pavlovic M., Perunicic M., Petrovic M., Mihaljevic B. (2009). Splenectomy with chemotherapy vs surgery alone as initial treatment for splenic marginal zone lymphoma. *World Journal of Gastroenterology*.

[18] Cervetti G., Galimberti S., Pelosini M., Ghio F., Cecconi N., Petrini M. (2013). Significant efficacy of 2-chlorodeoxyadenosine± rituximab in the treatment of splenic marginal zone lymphoma (SMZL): extended follow-up. *Annals of Oncology*.

[19] Else M., Marín-Niebla A., de la Cruz F., Batty P., Ríos E., Dearden C. E., Catovsky D., Matutes E. (2012). Rituximab, used alone or in combination, is superior to other treatment modalities in splenic marginal zone lymphoma. *British Journal of Haematology*.

